# Maternal smoking around birth as a risk factor for offspring chronic obstructive pulmonary disease: Evidence from Mendelian randomization

**DOI:** 10.18332/tid/203186

**Published:** 2025-04-29

**Authors:** Qiliang Jian, Huyan Huo, Fangxiang Mu, Fang Wang

**Affiliations:** 1 Department of Reproductive Medicine, Lanzhou University Second Hospital, Lanzhou, People’s Republic of China

**Keywords:** maternal smoking around birth, chronic obstructive pulmonary disease, Mendelian randomization, lung development

## Abstract

**INTRODUCTION:**

Previous observational studies suggested that exposure of the fetus to maternal smoking during pregnancy may increase the likelihood of chronic obstructive pulmonary disease (COPD). Hence, following the STROBE-MR guidelines, we carried out a two-sample MR analysis to explore the association between maternal smoking around birth and offspring COPD.

**METHODS:**

We used independent single nucleotide polymorphisms (SNPs) related to maternal smoking around birth, obtained from genome-wide association study summary data, as instrumental variables (IVs). The dataset included 121634 controls and 276098 cases. The selected outcome was chronic obstructive pulmonary disease (COPD) in offspring, with 454945 controls and 13530 cases. We performed analyses using inverse variance weighting (IVW), weighted median, and MR-Egger regression methods. Multivariate MR included maternal illnesses (high blood pressure and heart disease) as covariates to address potential mediators. Sensitivity analysis was conducted using leave-one-out analysis, Cochran's Q test, and MR-Egger intercept test.

**RESULTS:**

In the univariate MR analysis, it was found that maternal smoking around birth significantly increased the likelihood of offspring COPD (IVW, OR=35.13; 95% CI: 10.18–121.20; p<0.001). Furthermore, after adjusting the variates for maternal illnesses, the relationship between maternal smoking around birth and offspring COPD remained statistically significant (adjusted odds ratio, AOR= 62.11; 95% CI: 16.60–232.46; p<0.001).

**CONCLUSIONS:**

The study provides MR evidence of a potential association between maternal smoking around birth and increased COPD risk in offspring.

## INTRODUCTION

Chronic obstructive pulmonary disease (COPD) is a complex respiratory condition characterized by persistent and progressive airflow obstruction, resulting from abnormalities in the airways (such as bronchitis and bronchiolitis) or alveoli (emphysema)^1^. COPD is a major global health concern with an estimated prevalence of 12.16% worldwide^2^. It is among the top three causes of death, claiming approximately 3.2 million lives annually^3^. The primary pulmonary symptoms of COPD include dyspnea, breathlessness, cough and sputum, and chest tightness^4^. Even for those with mild COPD, the symptoms can be significant^5^. COPD can also lead to various extrapulmonary effects, such as weight loss, malnutrition, and skeletal muscle dysfunction^6^. These comorbidities significantly reduce both the survival time and quality of life for affected individuals. Although the existing treatment methods for COPD cannot reverse the course of the disease, they can effectively alleviate the clinical symptoms of patients. Drug treatment mainly includes bronchodilators. For glucocorticoids and antibiotics, non-drug treatment includes oxygen therapy, non-invasive ventilation, pulmonary rehabilitation, and surgical treatment. While the present treatment methods for COPD cannot reverse the course of the disease, they can effectively alleviate the clinical symptoms of patients^7^. However, there is still a pressing need to develop new and effective treatments to halt the decline in lung function in COPD patients. Encouragingly, research suggests that stem cell therapy may hold promise in improving lung function^8^.

Smoking is a well-established independent risk factor for COPD. Smokers are 2.55 times more likely to develop COPD compared to non-smokers^2^, as tobacco smoke leads to excessive mucus secretion in the trachea, alveolar wall destruction, and pulmonary fibrosis^9^. Despite the widely publicized harm of smoking, about 1.7% of pregnant women smoke during pregnancy, resulting in an increased risk to their fetuses’ lung health^10,11^. McEvoy et al.^12^ demonstrated a significant decrease in the time to peak tidal expiratory flow to expiratory time ratio (TPTEF/ET) among infants born to smoking mothers. The Hayatbakhsh et al.^13^ observational study of 1185 males and 1224 females found that male offspring exposed *in utero* to maternal smoking exhibited significant reductions in forced expiratory volume (FEV_1_) and forced expiratory flow (FEF) into early adulthood^13^, while the impact was not observed in female offspring. Beyer et al.^14^ suggested an association between maternal smoking during pregnancy and adult offspring’s COPD in a cohort of 291 patients, and this study was conducted using a structured questionnaire, which had significant confounding factors. Although these studies imply that maternal smoking during pregnancy can have lasting detrimental effects on offspring lung health and potentially increase the risk of COPD, they are observational and therefore susceptible to confounding bias and heterogeneous results. There is still a lack of robust evidence demonstrating an association between maternal smoking during pregnancy and offspring COPD.

Mendelian randomization (MR) is a causal inference method that utilizes genetic variation. The fundamental principle of MR is to infer the causal association of an exposure on an outcome using the effects of randomly assigned genotypes on phenotypes observed in natural populations^15^. As genetic variation is present at birth and remains stable throughout the life cycle, the resulting association obtained by MR is not affected by reverse causation or confounders^16^. Considering the limited evidence for an association between maternal smoking during pregnancy and offspring COPD, our study aimed to investigate this association using MR.

In this study, we adopted a two-sample MR approach to analyze the summary data from the genome-wide association study (GWAS) database. Based on the three assumptions of MR, we assessed the potential association between maternal smoking around birth as the exposure variable and offspring COPD as the outcome variable, following the STROBE-MR guidelines for conducting and reporting MR studies.

## METHODS

### Study design and data sources

**Figure 1:**
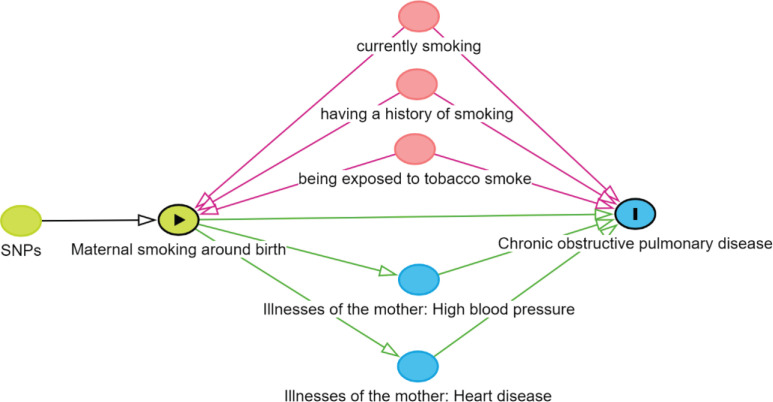
Directed acyclic graph (DAG) illustrating the univariate and multivariate MR analysis framework. Maternal smoking around birth is the exposure, and COPD in offspring is the outcome. The pink-colored nodes represent confounders. The blue-colored nodes represent mediators

This study conducted a two-sample MR analysis based on summary-level data, adhering to three main assumptions: 1) Relevance: genetic variations must be strongly associated with the exposure; 2) Independence: genetic variations should be independent of potential confounders; and 3) Exclusion restriction: genetic variations influence the outcome solely through the exposure, without alternative pathways (Supplementary file Figure S1). A directed acyclic graph (DAG) was drawn to illustrate the MR study design (Figure 1). The GWAS data used in the study were downloaded from the Integrative Epidemiology Unit (IEU) open GWAS project^17^. Developed by the MRC IEU at the University of Bristol, this public access, manually curated database complies summary statistics encompassing hundreds of billions of genetic associations from over 50000 complete GWAS datasets. These data represent different human genotypes and disease outcomes across diverse populations, enabling researchers to select appropriate genetic variations as instrumental variables (IVs), assess their association strength with exposure variables, and evaluate the potential risk of pleiotropy.

The exposure variable in this study was maternal smoking around birth, while COPD served as the outcome variable. To assess the influences of potential mediators, we included a covariate for the multivariate MR analysis, which consisted of illnesses of the mother, such as high blood pressure and heart disease. Maternal smoking around birth, maternal high blood pressure, and maternal heart disease were assessed through self-reported questionnaires, while COPD was primarily identified using electronic medical records. Summary data for maternal smoking around birth involved 397732 participants (121634 controls and 276098 cases). For COPD data, there were 468475 participants, consisting of 454945 controls and 13530 cases. The data for maternal high blood pressure included 426391 participants, with 130948 controls and 295443 cases. The data for maternal heart disease involved 426240 participants, with 85620 controls and 340620 cases (Table 1). The COPD GWAS dataset was sourced from the UK Biobank and FinnGen databases, and the remaining datasets were derived from the UKB database. All participants are of European descent.

**Table 1:** Brief description of the genome-wide association study data used

*GWAS ID*	*Year*	*Trait*	*Consortium*	*Sample size*	*Case*	*Control*	*nSNPs*
ukb-b-17685	2018	Maternal smoking around birth	MRC-IEU	397732	121634	276098	9851867
ukb-b-18167	2018	Illnesses of the mother: high blood pressure	MRC-IEU	426391	130948	295443	9851867
ukb-b-12477	2018	Illnesses of the mother: heart disease	MRC-IEU	426240	85620	340620	9851867
ebi-a-GCST90018807	2021	Chronic obstructive pulmonary disease	NA	468475	13530	454945	24180654

nSNPs: number of SNPs. NA: not applicable.

### Ethics

This study utilized publicly available GWAS summary data, for which the original studies had obtained ethical committee approval. No additional ethical approval or informed consent was required.

### Data collation

To collate the data, we selected independent single nucleotide polymorphisms (SNPs) associated with maternal smoking around birth and maternal diseases as IVs, which had genome-wide significance, from the database. This selection process aimed to identify strongly associated variations. Strong linkage disequilibrium (LD) among the SNPs was excluded from the analysis to avoid the bias of IVs. The screening criteria were as follows: 1) determining the genome-wide significance of maternal diseases and living habits based on the genome-wide information of the 1000 Genomes Project (p<5×10^-8^); 2) ensuring a physical distance of more than 10000 kb between every two genes; and 3) setting an R^2^ threshold of LD between genes to be <0.001. The selected IVs were analyzed for associations with other phenotypes using the PhenoScanner^18^, defined as having a history of smoking, currently smoking, and being exposed to tobacco smoke and air pollution in the offspring, to further reduce the possible impact of horizontal pleiotropy on the study. The following SNPs associated with these phenotypes were excluded: rs10226228, rs2183947, rs576982, rs6011779, rs75596189, and rs7899608. See Table 2 for the details of selected IVs.

**Table 2:** Details for SNPs

*Position*	*SNP*	*Other allele*	*Effect allele*	*EAF*	*β*	*SE*	*p*	*R^2^*	*F*
rs12405972	T	G	-0.00806153	0.001	0.000	-0.022	0.0122	0.0707294	56.247
rs12923476	A	G	-0.00686495	0.001	0.000	-0.036	0.0142	0.0122200	34.260
rs1323341	G	A	-0.00682946	0.001	0.000	-0.008	0.0141	0.5802000	30.203
rs2428019	A	C	0.00702278	0.001	0.000	0.002	0.0137	0.8888000	34.158
rs35566160	G	A	0.00637328	0.001	0.000	0.039	0.0126	0.0018940	29.927
rs36072649	A	T	-0.00717316	0.001	0.000	-0.047	0.0119	0.0000730	46.093
rs4865667	T	C	-0.00581087	0.001	0.000	-0.019	0.0121	0.1159000	30.451
rs62477310	C	T	-0.00577877	0.001	0.000	-0.018	0.0113	0.1090000	31.495
rs7002049	C	T	0.0075623	0.001	0.000	0.021	0.0159	0.1845000	36.620

SNPs that were used as instrumental variables met the conditions: p<5×10^-8^ and F>10. SE: standard error. SNP: single nucleotide polymorphism. EAF: effect allele frequency.

### Statistical analysis

The primary analysis methods used to evaluate the association between maternal smoking around birth and maternal diseases on childhood COPD were inverse variance weighting (IVW), MR-Egger regression, and weighted median (WM). IVW is the primary method. It applies weights based on the inverse variance of each IV, assuming the validity of all IVs, but is sensitive to pleiotropy. IVW does not include an intercept term in the regression, and the final causal estimate represents the weighted average of the effect values from all IVs. The key distinction between the MR-Egger method and IVW lies in the MR-Egger method’s incorporation of an intercept term to adjust for pleiotropy. This method relies on the Instrument Strength Independent of Direct Effect (InSIDE) assumption, which stipulates that the pleiotropic effects of IVs should be independent of their effects on the exposure. Furthermore, ME-Egger employs the inverse of outcome variance as the fitting weight. WM calculates the median of the weighted empirical density function, providing a consistent causal estimate even when IVs are invalid, as long as at least half the weight comes from valid IVs. These three methods are often combined to minimize bias due to pleiotropy or weak instrument strength.

The causal estimate from a two-sample analysis, where data on the risk factor and outcome are obtained from separate, non-overlapping datasets, is less prone to bias, with any residual bias tending toward the null. However, when utilizing genetic consortia that include partially overlapping participant sets, the direction and magnitude of bias become uncertain. Therefore, considering that the dataset used in the study includes overlapping samples, we employed the online tool ‘Bias and Type 1 error rate for Mendelian randomization with sample overlap’ to assess bias and Type 1 error rate^19^.

Multivariate MR methods were employed to adjust for covariates, specifically the mother’s illnesses of high blood pressure and heart disease. Other potential risk factors, such as active smoking, passive smoking, and air pollution, were not included in the adjustment, as SNPs related to these factors were excluded during the IV selection. In multivariate MR, the SNPs utilized as IVs must satisfy the following conditions: 1) they should be associated with all exposure factors in the model, 2) they should not impact the outcome variables through alternative pathways, and 3) the number of SNPs should exceed the number of exposure factors.

We conducted sensitivity analyses to evaluate the robustness of the results. Leave-one-out analysis test was used to assess the stability of individual SNPs by sequentially excluding each IV. Cochran’s Q test was applied to identify heterogeneity, with p<0.05 representing significant heterogeneity. MR-Egger intercept test was used to assess horizontal pleiotropy, with p<0.05 indicating significant horizontal pleiotropy.

All MR analyses were carried out using the R software. IVW, WM, and MR-Egger methods were implemented with the R package *Two Sample MR*. Multivariate MR methods were performed using the R package *Mendelian Randomization*. The evaluation indices used were odds ratio (OR) and 95% confidence interval (CI). A two-sided statistical significance level of p<0.05 was considered to indicate statistical significance. This study adhered to the STROBE-MR guidelines, with a detailed checklist provided in the Supplementary file.

## RESULTS

### Results of univariate MR

**Figure 2:**
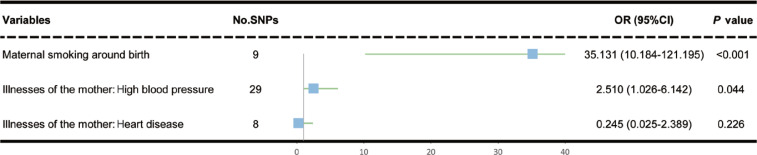
Univariate MR estimated the association of maternal smoking around birth, maternal high blood pressure, and maternal heart disease on COPD in offspring

Based on the IVW test results, it was found that maternal smoking around birth (IVW, OR=35.13; 95% CI: 10.18–121.20; p<0.001) and illnesses of the mother with high blood pressure (IVW, OR=2.51; 95% CI: 1.03–6.14; p=0.044) were significantly associated with an elevated risk of offspring COPD (Figure 2). However, there was no statistically significant association between illnesses of the mother with heart disease and COPD in the IVW test results (IVW, OR=0.25; 95% CI: 0.03–2.39, p=0.226). Similar findings of the relationship between maternal smoking around birth and offspring COPD were noted in the WM method (p<0.001) (Table 3).

**Table 3:** Heterogeneity and horizontal pleiotropy estimation

*Exposure*	*nSNPs*	*Method*	*β*	*SE*	*OR (95% CI)*	*p*	*FDR* *p*	*Cochran’s Q*			*MR-Egger*			*Egger intercept*	
								*Q*	*Q_df*	*p*	*Q*	*Q_df*	*p*	*Intercept*	*p*
Maternal smoking around birth	9	Inverse variance weighting (fixed effects)	3.559	0.632	35.13 (10.18–121.20)	**<0.001**	**<0.001**	10.374	8	0.240	10.267	7	0.174	0.013	0.795
		MR Egger	1.686	6.968	5.40 (0.00–4604595.09)	0.816									
		Weighted median	2.946	0.895	19.04 (3.30–109.98)	**<0.001**									
Illnesses of mother: High blood pressure	29	Inverse variance weighting (multiplicative random effects)	0.920	0.457	2.51 (1.03–6.14)	**0.044**	**0.029**	50.250	28	0.006	45.902	27	0.013	-0.023	0.121
		MR Egger	3.948	1.945	51.82 (1.15–2344.28)	0.052									
		Weighted median	0.703	0.508	2.02 (0.75–5.46)	0.166									
Illnesses of mother: Heart disease	8	Inverse variance weighting (multiplicative random effects)	-1.407	1.162	0.25 (0.03–2.39)	0.226	0.226	31.316	7	<0.001	28.176	6	<0.001	-0.014	0.445
		MR Egger	-0.318	1.787	0.73 (0.02–24.14)	0.864									
		Weighted median	-0.776	0.6746	0.46 (0.12–1.73)	0.250									

nSNPs: number of SNPs. SE: standard error. FDR: false discovery rate.

No horizontal pleiotropy was observed in our MREgger intercept results (intercept=0.013, p=0.795). According to Cochrane’s Q test (p=0.24), there was no evidence of heterogeneity between maternal smoking around birth and COPD. These results also reduce concerns about the InSIDE assumption. However, heterogeneity was found in Cochrane’s Q test and MR-Egger for the associations between high blood pressure and heart disease and COPD.

**Figure 3:**
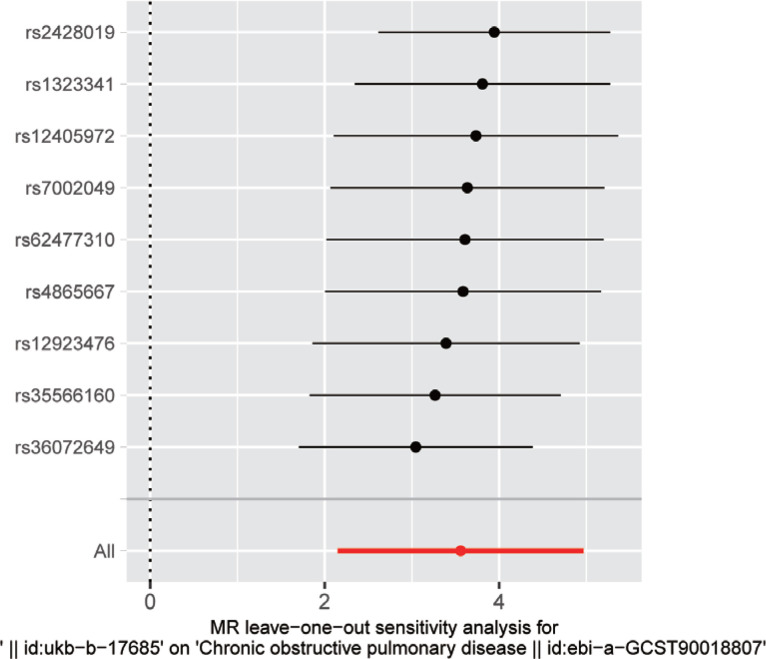
MR leave-one-out sensitivity analysis of maternal smoking around birth on COPD in offspring

The leave-one-out sensitivity analysis results indicated that upon removing each SNP one-by-one, the IVW analysis results of the remaining SNPs closely matched the analysis results obtained from including all SNPs (Figure 3, and Supplementary file Figures S2 and S3). This suggested that none of the SNPs had a strong effect on the estimated relationship.

We assessed bias and Type 1 error rate using the online tool. The results showed that even with complete sample overlap, the bias and Type 1 error rate remained at 0.005 and 0.05, respectively.

### Results of multivariate MR

**Figure 4:**
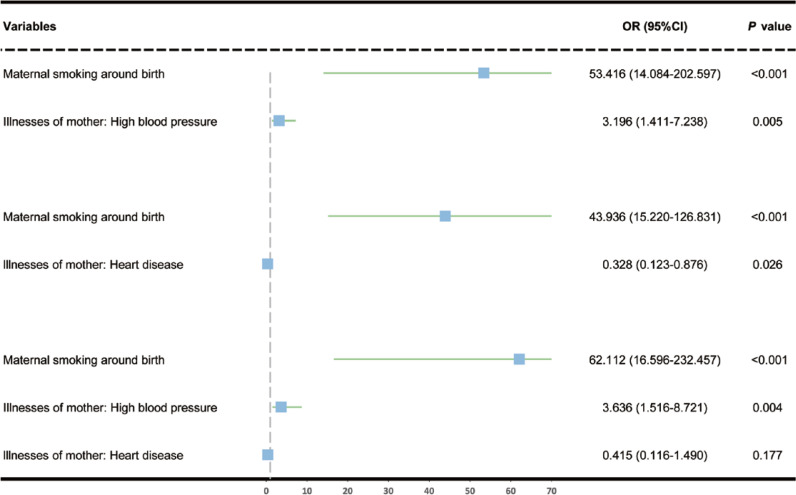
After adjustment for maternal high blood pressure and heart disease, both individually and together, multivariate MR estimated the association of maternal smoking around birth on COPD in offspring

After conducting multivariate MR adjustment for covariates, including maternal high blood pressure and maternal heart disease, the association between maternal smoking around birth and offspring COPD remained statistically significant (AOR=62.11; 95% CI: 16.60–232.46; p<0.001) (Figure 4 and Table 4). This suggested that even after accounting for these covariates, maternal smoking around birth is independently associated with an increased risk of offspring COPD. Notably, this multivariate MR analysis did not adjust for active smoking, passive smoking, and air pollution because SNPs related to these factors were excluded during IV selection.

**Table 4:** Multivariable Mendelian randomization results of maternal smoking around birth on COPD

*Exposure*	*nSNPs*	*β*	*SE*	*AOR (95% CI)*	*p*
Maternal smoking around birth	9	3.978	0.680	53.42 (14.08–202.60)	**<0.001**
Illnesses of mother: High blood pressure	27	1.162	0.417	3.20 (1.41–7.24)	**0.005**
Maternal smoking around birth	10	3.783	0.541	43.94 (15.22–126.83)	**<0.001**
Illnesses of mother: Heart disease	7	-1.115	0.501	0.33 (0.12–0.88)	0.026
Maternal smoking around birth	9	4.129	0.673	62.11 (16.60–232.46)	**<0.001**
Illnesses of mother: High blood pressure	27	1.291	0.446	3.64 (1.52–8.72)	**0.004**
Illnesses of mother: Heart disease	6	-0.879	0.652	0.42 (0.12–1.49)	0.177

nSNPs: number of SNPs. SE: standard error. COPD: chronic obstructive pulmonary disease. AOR: adjusted odds ratio. The multivariate MR analysis adjusted for maternal high blood pressure and maternal heart disease. SNPs associated with active smoking, passive smoking, and air pollution were excluded from instrumental variable selection, and thus these factors were not adjusted for.

## DISCUSSION

In this MR analysis, both univariate and multivariate IVW results consistently suggested a relationship between maternal smoking around birth and an increased risk of COPD in offspring. These findings provide complementary evidence to previous observational studies. For example, Beyer et al.^14^ reported the influence of maternal smoking during pregnancy or after delivery on the predisposition of offspring to COPD in adulthood in a German population (n=291). The Li et al.^20^ case-control study suggested a significant direct effect of maternal smoking during pregnancy on offspring COPD in a Chinese population (n=5943). The Upton et al.^21^ study found that offspring of mothers who smoked ≥25 cigarettes per day had a six-fold increased risk of developing COPD compared to those unexposed to maternal smoking during pregnancy in a British population (n=3202). However, due to ethical considerations, these studies are cross-sectional or retrospective, making it difficult to establish the chronological order of exposure and outcome. Thus, they may be subject to reverse causality, confounding bias (e.g. maternal health, environmental factors), selection bias (related to specific population), and recall bias (inaccurate self-reported maternal smoking history). The relatively small sample sizes (usually in the hundreds or thousands) can also limit statistical power and precision. In contrast, our MR analysis employed SNPs as IVs to mitigate these biases and yielded more accurate estimates of potential causality, leveraging a much larger GWAS sample of European ancestry (COPD, n=468475). Furthermore, MR studies leverage the random allocation of genetic variation, akin to the design of a randomized controlled trial, thereby strengthening causal inference beyond mere association. A previous MR study^22^ suggested an association between maternal smoking around birth and offspring COPD (6915 cases and 186723 controls), using SNPs associated with exposure at p<5.0×10^-7^. Our MR analysis provides more robust evidence for this association. We utilized a larger outcome dataset (13530 cases and 454945 controls) and employed a more stringent SNP selection threshold of p<5.0×10^-8^. Moreover, we adjusted for offspring active smoking and passive tobacco exposure, providing stronger support for an association between maternal smoking around birth and an increased risk of offspring COPD.

Maternal smoking during pregnancy is widely recognized to impair respiratory function in offspring during infancy or childhood^23,24^. The Gilliland et al.^25^ observational study evaluated 5762 school-aged children across 12 American communities. The study concluded that maternal smoking during pregnancy heightened the risk of physician-diagnosed asthma and wheezing in childhood, while exposure to environmental tobacco smoke was considered a trigger, rather than a factor of wheezing^25^. Additionally, another observational study involving 5951 children aged 8–12 years from 9 Russian cities established a strong association between prenatal exposure to maternal smoking and conditions such as asthma, chronic bronchitis, and respiratory symptoms, though early and current exposures showed a weaker link to these diseases^26^.

Lung development occurs in three stages, namely embryonic, fetal, and alveolar. Any disruption during these stages can significantly impact respiratory system development^27^. The potential mechanisms by which maternal smoking during pregnancy leads to health problems in offspring from the many toxicants in tobacco smoke include *in utero* hypoxia, nicotine-induced reductions in uteroplacental blood flow, placental toxicity, and toxic growth restriction^28^. The harmful effects of tobacco smoke are primarily mediated by the release of carbon monoxide and nicotine. Carbon monoxide can diffuse through the placental barrier and enter the fetal circulation, binding with fetal hemoglobin, and leading to an increase in carboxyhemoglobin. This causes a leftward shift in the oxygen dissociation curve, thereby reducing the oxygen released to fetal tissues, resulting in fetal hypoxia which is detrimental to the development of the lungs and brain^29,30^. Additionally, prenatal exposure to nicotine has been validated in numerous animal models to impair fetal lung development. Experiments conducted on rhesus monkeys have demonstrated that prenatal exposure to nicotine increases the size of fetal pulmonary vascular walls and enhances the expression of collagen and elastin in airway vessels^31^. These changes alter the mechanical properties of the pulmonary vasculature and result in impaired lung development. Wongtakool et al.^32^ found that prenatal exposure to nicotine in fetal mice significantly reduces forced expiratory flow after birth and increases the deposition of collagen proteins in airways and blood vessels. These findings suggest that nicotine exposure leads to an increase in airway length and a decrease in diameter, resulting in reduced lung function^32^. Similarly, Sandberg et al.^33^ observed similar findings in lambs, where fetal exposure to nicotine resulted in increased smooth muscle volume but decreased bronchial diameter, thereby limiting airflow during inhalation. Furthermore, other studies have suggested that prenatal exposure to nicotine significantly increases lung and parenchyma volumes, as well as septal surface area after birth^34^. Collectively, these studies suggest that fetal exposure to tobacco during pregnancy impairs fetal lung and tracheal development, leading to respiratory abnormalities. This may potentially explain the increased risk of COPD among offspring due to maternal smoking around birth. However, due to limited evidence of randomized control trials (RCTs), further research is needed to establish the precise link and underlying mechanisms.

### Strengths and limitations

This study provides MR evidence of the relationship between maternal smoking around birth and the risk of COPD in offspring. The results maintain their robustness due to the large scale of the sample size drawn from the databases, absence of pleiotropy, and the elimination of confounders associated with the outcome, such as personal smoking and environmental factors. We further enhanced the transparency of our research process by using online tools to calculate the bias and Type 1 error rate introduced by sample overlap, thereby increasing the reproducibility and credibility of our findings.

However, there are several limitations to consider. Firstly, there was population overlap in our analysis dataset, which could potentially bias the estimated association between maternal smoking around birth and offspring COPD. Secondly, the GWAS data used in this study were derived exclusively from individuals of European ancestry, which may compromise the generalizability of the findings to non-European populations. Genetic associations may vary across populations due to potential differences in allele frequencies, LD patterns, and gene-environment interactions^35,36^, which we were unable to assess in this study. Future studies incorporating diverse ancestral backgrounds are needed to enhance the applicability of the results. Thirdly, although MR analysis minimizes confounding by observed factors, we cannot entirely rule out residual confounding due to unmeasured or unknown confounding factors. Fourthly, although we performed sensitivity analyses to assess for potential pleiotropy, these methods have limited power to detect and correct for all forms of pleiotropy, especially when the pleiotropic effects are balanced or acting in opposing directions. This may violate the MR assumptions and influence the causal estimates. Fifthly, it is challenging to determine the exact number of cigarettes smoked by pregnant women, which may lead to an overestimation of the results among pregnant women who smoke only a limited amount. Nonetheless, given the health risks associated with smoking during pregnancy, any form of smoking should be avoided. Finally, while this study suggests an association between maternal smoking and an increased risk of COPD with genetic variations, it should be noted that MR analysis is primarily a statistical tool and requires additional support from future clinical studies.

## CONCLUSIONS

Our study presents MR evidence supporting an association between maternal smoking around birth and the increased risk of offspring COPD in European populations. This finding indicates maternal smoking as an independent risk factor for offspring COPD.

## Supplementary Material



## Data Availability

The data supporting this research are available from the following source: https://gwas.mrcieu.ac.uk/datasets (IEU Open GWAS).
