# Supplementary material for: Maternal smoking around birth as a risk factor for offspring chronic obstructive pulmonary disease: Evidence from Mendelian randomization
Source: Tob Induc Dis. 2025 Apr 29;23:10.18332/tid/203186. doi: 10.18332/tid/203186 (PMC12039807; doi:10.18332/tid/203186)
Supplement: Supplementary file 1 [file TID-23-51-s1.pdf]

Supplementary materials

Figure S1 Fundamental assumptions of MR.

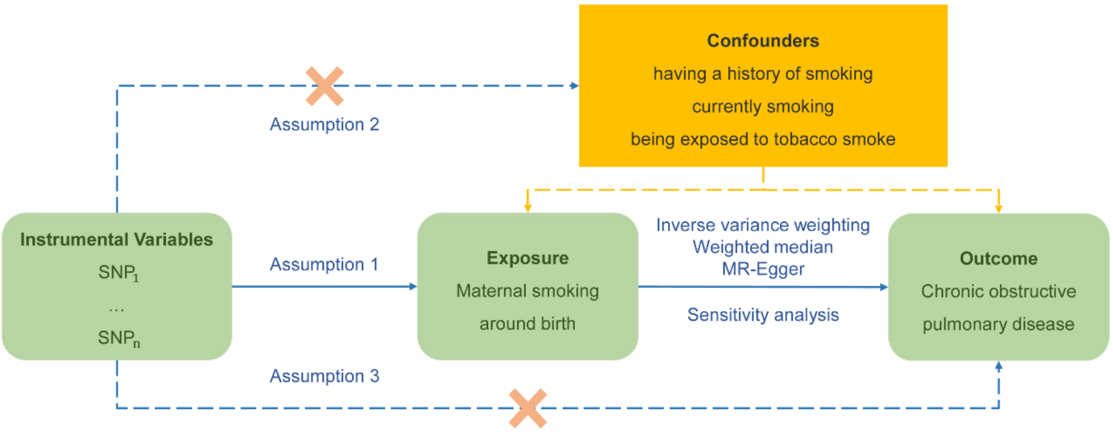

Assumption 1: Genetic variations must be strongly associated with the exposure; Assumption 2: Genetic variations should be independent of potential confounders; (3) Assumption 3: Genetic variations influence the outcome solely through the exposure, without alternative pathways

Figure S2 MR leave-one-out sensitivity analysis of maternal high blood pressure on COPD in offspring.

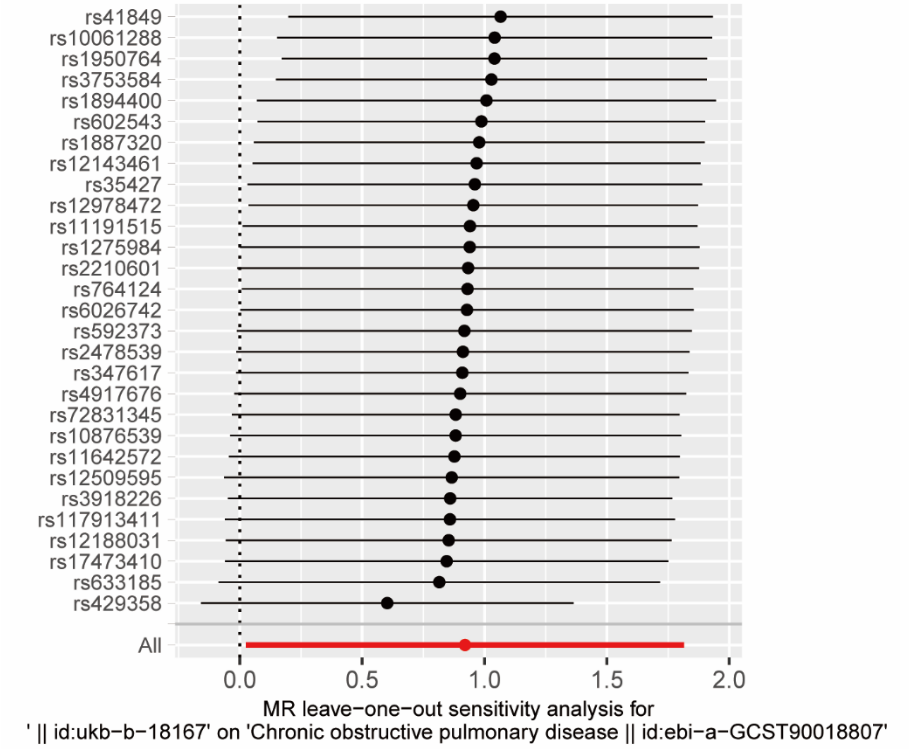

Figure S3 MR leave-one-out sensitivity analysis of maternal heart disease on COPD in offspring.

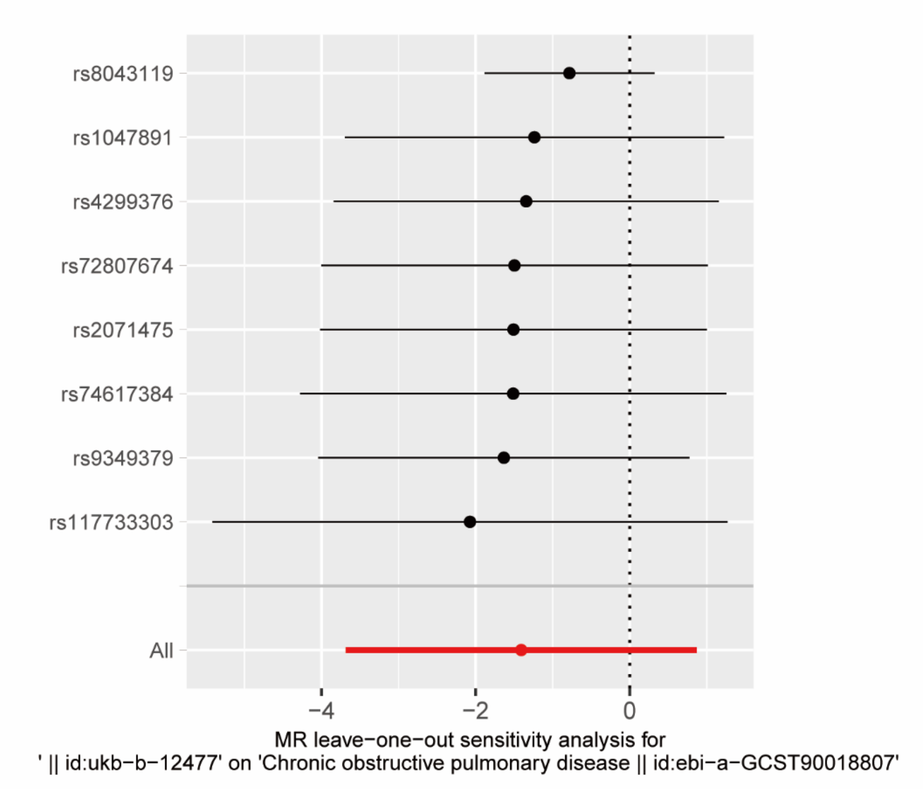

© 2025 Jian Q. et al.
